# Inclusion of a Catechol-Derived Hydrazinyl-Thiazole (CHT) in β-Cyclodextrin Nanocavity and Its Effect on Antioxidant Activity: A Calorimetric, Spectroscopic and Molecular Docking Approach

**DOI:** 10.3390/antiox12071367

**Published:** 2023-06-29

**Authors:** Mihaela Mic, Adrian Pîrnău, Călin G. Floare, Mariana Doina Palage, Ovidiu Oniga, Gabriel Marc

**Affiliations:** 1 National Institute for Research and Development of Isotopic and Molecular Technologies, 67-103 Donat, 400293 Cluj-Napoca, Romania; mihaela.mic@itim-cj.ro (M.M.); calin.floare@itim-cj.ro (C.G.F.); 2 Department of Therapeutical Chemistry, “Iuliu Hațieganu” University of Medicine and Pharmacy, 12 Ion Creangă Street, 400347 Cluj-Napoca, Romania; mpalage@umfcluj.ro; 3 Department of Pharmaceutical Chemistry, “Iuliu Hațieganu” University of Medicine and Pharmacy, 41 Victor Babeș Street, 400012 Cluj-Napoca, Romania; ooniga@umfcluj.ro (O.O.); marc.gabriel@umfcluj.ro (G.M.)

**Keywords:** catechol, antioxidant, thiazole, cyclodextrin, ITC, NMR, molecular docking

## Abstract

The aim of the present research was to obtain a supramolecular complex between a strong antioxidant compound previously reported by our group, in order to extend its antioxidant activity. The formation of the inclusion complex of a catechol hydrazinyl-thiazole derivative (CHT) and β-cyclodextrin in aqueous solution has been investigated using isothermal titration calorimetry (ITC), spectroscopic and theoretical methods. The stoichiometry of this inclusion complex was established to be equimolar (1:1) and its equilibrium constant was determined. An estimation of the thermodynamic parameters of the inclusion complex showed that it is an enthalpy and entropy-driven process. Our observations also show that hydrophobic interactions are the key interactions that prevail in the complex. ^1^H NMR spectroscopic method was employed to study the inclusion process in an aqueous solution. Job plots derived from the ^1^H NMR spectral data demonstrated 1:1 stoichiometry of the inclusion complex in a liquid state. A 2D NMR spectrum suggests the orientation of the aromatic ring of CHT inside the β-CD cavity. The antiradical activity of the complex was evaluated and compared with free CHT, indicating a delayed activity compared with free CHT. To obtain additional qualitative and visual insight into the particularity of CHT and β-CD interaction, molecular docking calculations have been performed.

## 1. Introduction

Nowadays, the inclusion of compounds in cyclodextrins (CDs) is a common approach to optimize the properties of the incorporated compounds such as solubility, stability, reduction in tissue irritation, odor, or taste masking in the field of food, cosmetics, pharmaceutics, agriculture, or wastewater treatment [1,2,3,4]. Due to their low toxicity, this field attracted the interest of researchers for years before and we can assume that the interest of obtention of various supramolecular complexes with CD will be of interest in the next years [5].

**Figure 1:**
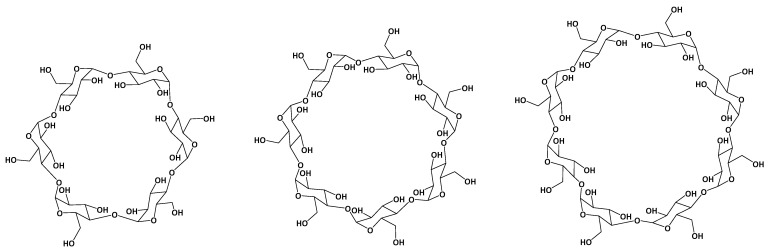
Chemical structure of α-CD (**left**), β-CD (**center**), and γ-CD (**right**).

Cyclodextrins are macrocyclic oligosaccharides comprised of 6, 7, or 8 units of 1,4-linked glycosidic α-D-glucopyranose units, named α, β, and γ-cyclodextrins, respectively [6]. The chemical structures of α-CD, β-CD, and γ-CD are presented in Figure 1. The shape of the CDs is described in the literature in many ways, from a doughnut to a truncated cone [3,7], while the inner cavity has a cone-like shape [4]. The diameter of the inner cavity increases with the number of glucose units from 0.78 nm for α-CD, 0.78 nm for β-CD, and 0.95 nm for γ-CD, respectively [1].

CDs are amphiphilic—the internal cavity of the cyclodextrins is hydrophobic, where various hydrophobic small guest compounds can accommodate, while the outside of CDs is hydrophilic favoring the interaction of the complex with water. Based on these properties, CDs are used for an increase in solubility in water of organic compounds used as drugs with low solubility [1,8,9].

The inclusion in the CD of interesting compounds resulting in the supramolecular complexes would act as a potential reservoir by trapping the compounds inside and releasing controlled kinetics [10]. This is linked with the way the guest compound interacts with CD, the main driving interactions between them are hydrophobic interactions: van der Waals forces, electrostatic interactions, and hydrogen bonds, all of them being weak, non-covalent bonds. CDs on the other hand, due to its property of encapsulating molecules that are hydrophobic in nature, are widely applied in the field of drug delivery [11].

Applying this principle in the field of antioxidants would allow CDs to be considered “secondary antioxidants” [12]. The inclusion in CD of strong polyphenolic antioxidants such as quercetin, rutin, catechin, epicatechin, brazilin, resveratrol, kaempferol, myricetin, naringenin, baicalein, gallates, and caffeic acid which are susceptible to degradation via oxidation or photodegradation was reported in the literature, with consistent research regarding the polyphenol–CD interactions [2,3,7,13,14,15,16,17,18,19]. Also, the encapsulation of CD of vegetal extracts with antioxidant properties rich in polyphenols was reported [3,20,21].

**Figure 2:**
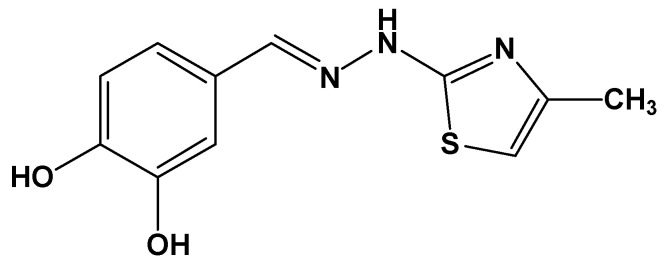
Chemical structure of CHT [22].

In our previous publication, we reported the obtention, characterization, and evaluation of a new phenolic compound linked via a hydrazine bridge to a thiazole ring (IUPAC: 4-((2-(4-methylthiazol-2-yl)hydrazineylidene)methyl)benzene-1,2-diol) with strong antioxidant and antiradical activity [22]. The chemical structure of CHT is presented in Figure 2. The present paper focuses on the inclusion of CHT in cyclodextrin to prolong the antioxidant activity in time, compared to the free state of the compound. This working hypothesis is based on our observation during the in vitro evaluation of the activity of CHT, that the scavenging of radicals used as reagents in the antiradical assays such as DPPH (2,2-diphenyl-1-picrylhydrazyl) and ABTS (2,2’-azino-bis(3-ethylbenzothiazoline-6-sulfonic acid) occurred at a very high speed. The endpoint of the reactions was reached much sooner than the time needed to complete the assays, according to the reports about protocols of performing the respective assays [22,23].

## 2. Materials and Methods

The reagents and solvents used for all activities presented in the research were purchased from local suppliers. For ITC and NMR measurements, we used β-CD from Sigma-Aldrich Chemie GmbH (St. Louis, MO, USA) and aqueous solutions were prepared in double distilled water and deuterium oxide, respectively.

### 2.1. Antioxidant Activity

The antioxidant activity of the β-CD complex of CHT was assessed using a modified protocol of DPPH scavenging assay, based on the initial report of Brand-Williams et al. [24,25,26]. The principle of the assay consists of the transfer of a hydrogen atom from the antioxidant compound to DPPH˙ (2,2-diphenyl-1-picrylhydrazyl). Following the binding of the hydrogen atom to the radicalized nitrogen atom, the resulting compound loses the intense purple color characteristic to the radical. The loss of the absorbance of the reagent at λ = 517 nm is proportional to the amount of DPPH˙ neutralized. Three measurements were made against a blank sample and the results are presented as averages, based on Equation (1). The absorbance of the samples was made using a UV-VIS spectrophotometer Jasco V-530 (Jasco International Co., Tokyo, Japan) in 10 mm single-use plastic cuvettes.

The stock solution of the stock CHT-β-CD complex was diluted using water at the final 250 µM concentration. Additionally, a solution of CHT 250 µM was obtained by dissolving solid CHT in water.

The working solution of the DPPH reagent was made by dissolving solid DPPH in methanol and later was diluted in water, resulting in a DPPH solution in 30% methanol and 70% water. The choice of this mixture of solvents was made to avoid the use of ethanol to avoid disruption of the CHT-β-CD complex. Similar solvent mixtures were reported in the literature for DPPH scavenging evaluation of β-CD complexes with other phenolic compounds [27,28]. Because DPPH is not soluble in water, methanol was chosen and the dilution of the initial methanolic solution with water was made to minimize the potential effect of methanol on the stability of the CHT-β-CD complex. The reagent absorbance at λ = 517 nm was made by dilution of the reagent solution with methanol:water 30:70 (*v*/*v*), until it reached approximatively A = 1.

In total, 50 µL of 62.5 µM, 125 µM, and 250 µM solutions of CHT-β-CD complex and CHT were mixed with 1 mL of DPPH reagent. The absorbance of the solutions at λ = 517 nm was measured every 30 s for 15 times, resulting in a total 450 s between the mixture of the samples with the working reagent and the last absorbance read.

(1) $$DPPH˙\;scavenging(\%)=\frac{control\;absorbance-sample\;absorbance}{control\;absorbance}\times 100$$

### 2.2. Isothermal Titration Calorimetry (ITC)

The binding between CHT and β-CD was investigated through ITC experiments carried out with a Nano ITC2G calorimeter (TA Instruments, New Castle, DE, USA) at a constant temperature of 25 °C. Prior to the titration experiments, both solutions were degassed under a vacuum for 30 min. The experiment consisted typically of injecting 10 μL of CHT ligand (4 mM) into the calorimetric cell that contains the β-CD solution (1 mM) by using a computer-controlled Hamilton micro-syringe. The reference cell was loaded with water. The interval of 300 s between injections was set to allow the heat signal to return to the baseline. During the titration, the reaction mixture was continuously stirred at 250 rpm to ensure proper mixing after each injection. The control experiment was performed by titrating CHT into a cell containing only water and the reference signal was subtracted from corresponding experimental data. The integrated heat data, after the correction for control, were analyzed using an independent binding model supplied by Nano Analyze 3.1.2 (TA Instruments, New Castle, DE, USA). The binding constant (K), the binding stoichiometry (*n*), the change in enthalpy (ΔH), and the change in entropy (ΔS) were thus obtained.

### 2.3. ^1^H NMR Analysis

NMR measurements were performed with Bruker AVANCE III spectrometer in a liquid state, operating at 500.13 MHz for protons which is equipped with a BBO (broad-band observe) probe head with Z-gradient. All samples were prepared in D_2_O solutions at 298 ± 0.1 K and all chemical shifts were measured relative to TMSP (3-(trimethyl-silyl)-propionic acid sodium salt), referencing the chemical shift to 0 ppm. The determination of the association constant and the stoichiometric ratio between CHT and β-CD by NMR, were made with two stock solutions in D_2_O, both having a concentration of 10 mM. Thus, 11 separate samples were prepared based on these two equimolar solutions which contain CHT and β-CD in a different ratio, by mixing the two stock solutions to constant volume in a complete interval (0 < r < 1) of the ratio r = [X]/([H] + [G]), where X = H or G and [H] and [G] are the total concentrations of the host (β-CD) and guest (CHT), respectively, with total concentration kept constant for each sample [H]_t_ + [G]_t_ = 10 mM.

^1^H NMR experiments were recorded using 64 scans collected into 65 K data points over a 5000 Hz spectral window, using a 4 s relaxation delay and an excitation pulse of 10.1 μs. The ROESY NMR spectrum was obtained using the pulse program *roesyphpr* with residual water suppression and the following acquisition parameters: 16 scans, 8192/2048 data points, 5000 Hz spectral width (10 ppm), spin-lock mixing time 350 ms.

### 2.4. Molecular Docking

**Figure 3:**
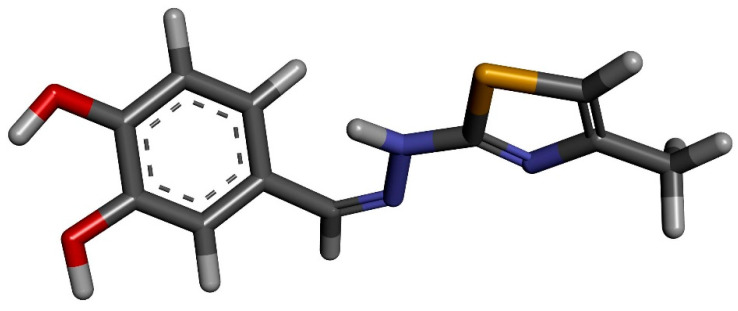
Gaussian 09 optimized molecular structure of CHT at the level M06-2X/6-311 ++ G(d,p).

The molecular docking of the CHT molecule to β-cyclodextrin has been performed following a protocol similar to that used in a preceding study where we analyzed the complexation of CHT and human serum albumin (HSA) [22]. The same molecular structure of CHT was used which was optimized using Gaussian 09 software [29] with density functional theory (DFT) based M06-2X Minnesota exchange-correlation energy functional which proved to perform better than B3LYP functional for model systems with dispersion and hydrogen-bonding interactions [30] and 6-311 ++ G(d,p) basis set. No imaginary frequencies were obtained. In Figure 3, the optimized structure of CHT is presented.

The β-cyclodextrin molecular structure was also the same as the ones used in some of our previous publications [31,32] and was extracted from a crystallographic inclusion compound with Mg^2+^ and Ca^2+^ salts of meclofenamic acid, determined by Caira et al. [33] (CSD entry: WERGUW).

A molecular docking calculation aim is to identify the preferred orientation of a smaller molecule (ligand or guest) to a large molecule or protein (receptor or host) and uses an empirical Monte Carlo simulated annealing search, estimating, in this way, the ligand affinity and activity. AutoDockTools v1.5.6 user interface of AutoDock v4.2 [34] was used to prepare the initial pdbqt files for the ligand and host molecule and the input files required by AutoDock [35]. While the atoms of the β-CD molecule were kept rigid, the flexibility of the ligand had been considered by setting up 5 torsion angles around rotatable bonds. AutoGrid was used in the precalculation of the grid maps of interaction energies of various atom types.

The search box with a cubic form and centered on the β-CD molecule, had a grid size of 60 × 60 × 60 points with a grid point spacing of 0.375 Å. All hydrogens atoms were added to the host molecule and the ligand structure was optimized including the hydrogens (as can be seen in Figure 2) but, during the preparation stage, the non-polar ones were automatically merged. The atomic charges were computed using the Gasteiger-Marsili method [36] implemented in AutoDockTools. The iterative generation and optimization of ligand conformations have been performed using the Lamarckian genetic algorithm [37,38]. We performed 2000 runs, each with a random generation seed, with the aim to obtain good statistics and optimized conformations clustering.

The analysis and visualization of the results, apart from AutoDockTools use, have been also realized with Chimera v1.14 [39] and Biovia Discovery Studio Visualizer v20.1.3.

## 3. Results

### 3.1. Antioxidant Activity

**Figure 4:**
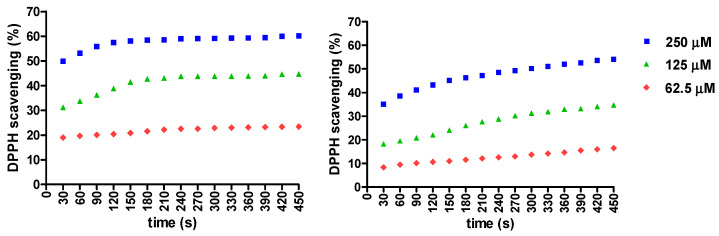
DPPH scavenging (%) of the CHT free (**left**) and CHT-β-CD complex (**right**) evaluated at three concentrations.

The antiradical activity of the CHT-β-CD complex was determined using the DPPH neutralization assay in comparison to free CHT, to evaluate how the complexation with β-CD influences its antiradical activity in time. The antiradical activity at the three concentrations of free CHT and CHT-β-CD complexes was evaluated, and their results are presented in Figure 4.

Free CHT reaches the endpoint of the DPPH scavenging reaction in approximately 150 s, after that the neutralization of the radical stays in a constant plateau. The final plateau value is dependent on the concentration of the sample involved in each assay.

On the other hand, the evaluation of the antiradical activity of the CHT-β-CD complex exhibits a quasi-linear inhibition of the DPPH reagent dependent on time. Compared with free CHT it can be observed that DPPH neutralization occurs in time, with sustained release of CHT from its β-CD complex. At the end of the assay (t_15_ = 450 s) the percentage of DPPH scavenged by the CHT-β-CD complex was lower than the CHT free, indicating that some of CHT was still sequestered in the β-CD complex at the end of assay, at all three concentrations evaluated.

### 3.2. Binding Affinity and Thermodynamics of CHT—β-CD Binding

Isothermal titration calorimetry (ITC) is the most sensitive and accurate analytical technique for the determination of binding constant and various thermodynamic parameters in macromolecule–bioligand complexation with precise accuracy [40,41,42,43].

**Figure 5:**
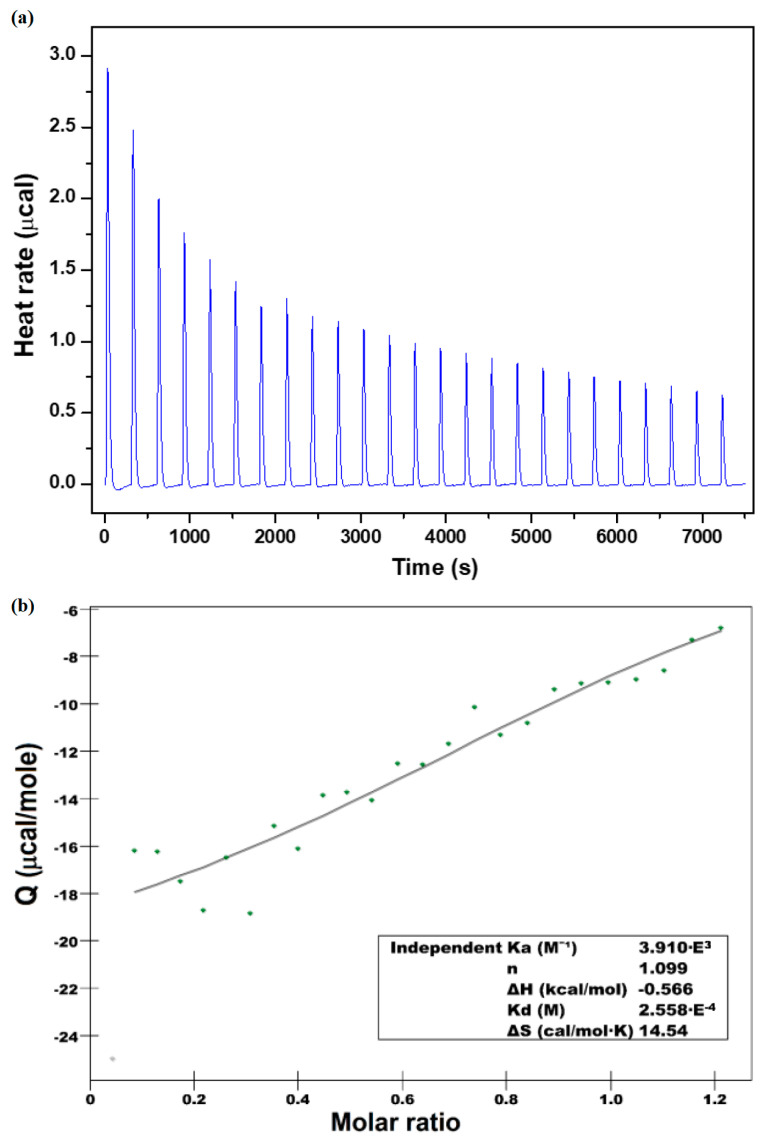
Isothermal titration calorimetry data obtained for the binding interaction in water at 25 °C of CHT (4 mM) to β-CD (1 mM). (**a**) Shows exothermic heat release upon injection of 10 μL aliquots of CHT into β-CD solution. (**b**) Shows integrated heat data. The solid line corresponds to the best-fit curve.

To evaluate the binding affinity and the thermodynamic profile of the inclusion complex of the CHT inside the β-CD in solution, isothermal calorimetry titration was carried out. To this effect, the representative calorimetric titration profile of the β-CD with CHT is depicted in Figure 5.

The binding isotherm (panel a) was obtained by monitoring the heat associated with injections. The magnitude of the released heat decreases progressively with which injection. The second quadrant of the figure shows the plot of the amount of heat release per injection as a function of the molar ratio of the drug to the β-CD (β-CD /CHT). An analysis of the binding isotherm using an independent binding model allowed for a description of the values of the binding and thermodynamic parameters (Table 1).

From the analysis of the thermodynamic data (Table 1), it can be seen that the stoichiometric ratio (*n*) which determines the number of β-CD molecules which includes the CHT molecules is *n* = 1.098. This may suggest that one mole of CHT molecule interacts with one mole of β-CD molecule. The equilibrium constant obtained from ITC measurement was K = 3.92 × 10^3^ M^−1^, which corresponds to a Gibbs free energy ΔG = −20.5 kJ/mol, indicating spontaneous inclusion of the drug into β-cyclodextrin.

The complexation process in the real system is exothermic, as confirmed by the negative change of the molar enthalpy value (ΔH =−2.36 kJ/mol) of the interaction between β-CD and CHT. The entropic effect of the binding process is TΔS = 18.13 kJ/mol and brings an additional contribution to the negative Gibbs free energy. The positive value of the entropy change is due to disturbing the ordered aqueous microenvironment surrounding the hydrophobic parts of the guest molecule after the binding to cyclodextrin. Furthermore, the enthalpic effect of binding for the discussed molecules has been dominated by the entropic effect of complex formation (|ΔH| < |TΔS|) and the releasing the water molecules from the cavity.

ITC data show that the encapsulation is both enthalpically and entropically driven, confirming once again that the hydrophobic interaction prevailing for this complex formation, which accounts for the negative sign of the enthalpy and the positive sign of the entropy changes.

### 3.3. Determination of the Stoichiometry by ^1^H NMR

^1^H NMR spectroscopy was used to highlight the formation in solution of the molecular complex CHT:β-CD, by including the CHT molecule in the hydrophobic cavity of β-CD. This fact is highlighted by changing the values of the chemical shifts of some protons of CHT and β-CD—those involved in this interaction, compared to the chemical shifts of the same protons in the free state. The determination of the molecular structure of the CHT was reported by our group in the paper of Mic et al. [22], where we find the total ^1^H NMR spectrum of the CHT molecule in D_2_O, with the assignment of the signals. Therefore, we will not resume this discussion and based on this assignment and the numbering of the protons in the CHT molecule presented in Figure 5, we summarize this information in Table 2, where it could be found the chemical shifts of the protons of the CHT molecule in the free state (in the absence of the β-CD).

**Figure 6:**
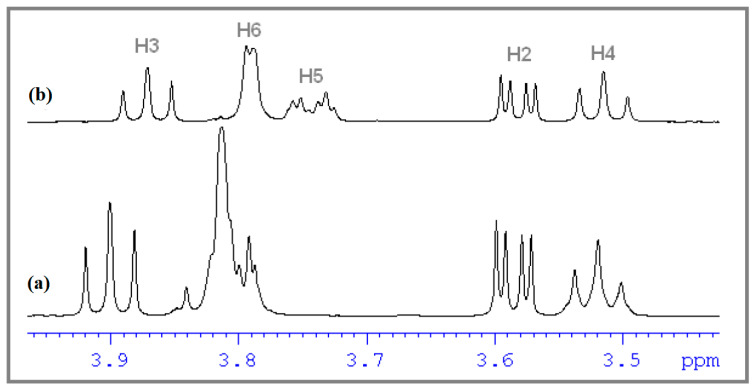
Partial ^1^H NMR spectrum of: (**a**) 10 mM β-CD; (**b**) 5 mM CHT and 5 mM β-CD.

**Figure 7:**
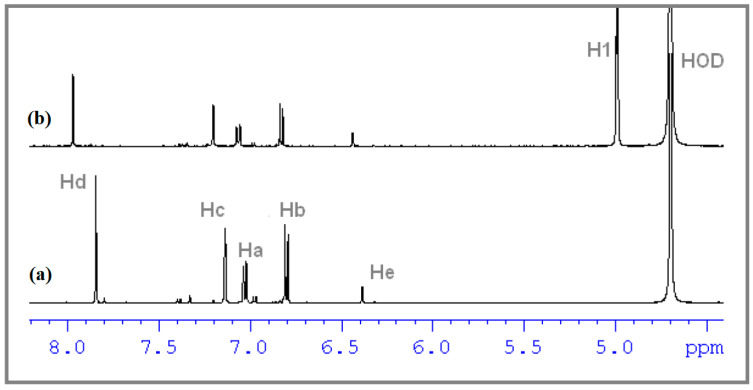
Partial ^1^H NMR spectrum of: (**a**) 10 mM CHT; (**b**) 5 mM CHT and 5 mM β-CD.

**Figure 8:**
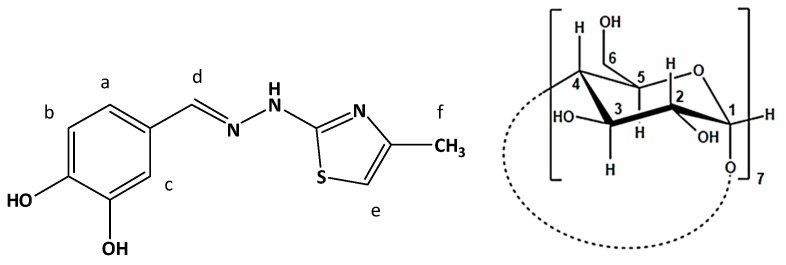
Chemical structure and atomic numbering of CHT (**left**) and β-CD (**right**).

Figure 6 and Figure 7 show ^1^H NMR spectra plotted in different molar ratios of CHT and β-CD, considering the numbering of the atoms of CHT and β-CD from Figure 8. Thus, the shifts of the signals specific to both CHT and β-CD molecules can be observed, compared to other signals that do not show changes in chemical shifts, including the residual proton signal from D_2_O.

**Figure 9:**
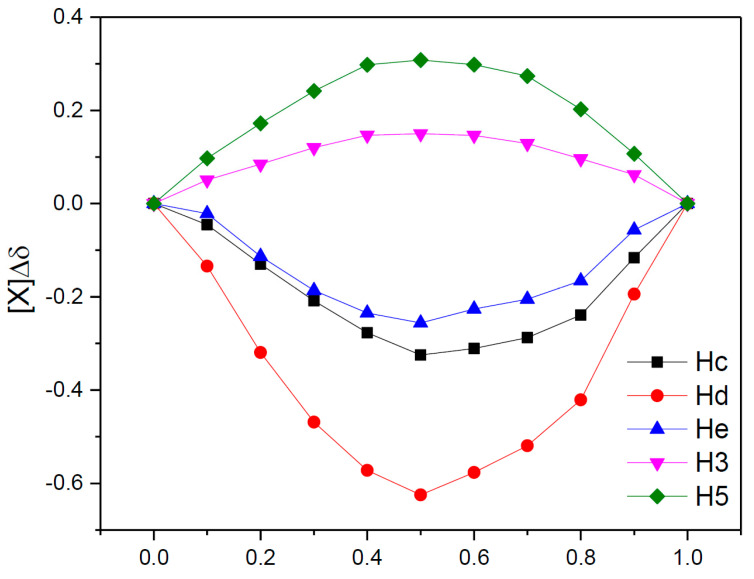
Concentrations variation plot for: H3 and H5 protons of β-CD; Hc, Hd, and He protons of CHT, where [X] = [CHT] or [β-CD].

As can be seen, in the spectra we have variations in the chemical shifts of some protons from the CHT molecule in the presence of β-CD. Also, the protons on the interior of the β-CD (H3 and H5) show a variation of the chemical shifts in the presence of CHT, which certifies an interaction between the CHT and the interior of the β-CD cavity, particularly the formation of the inclusion complex CHT:β-CD. The stoichiometry of the CHT:β-CD complex was determined using the continuous variation method, which involves the induced chemical shift, Δδ, which is dependent on the concentration of the complex [44]. Since the continuous variation graphs (Figure 9) have a symmetrical shape, with r having a maximum value of 0.5, we can assume that the inclusion complex has a stoichiometry of 1:1. This was highlighted both for protons belonging to the CHT molecule, and for H3 and H5 belonging to the interior of the β-CD cavity.

### 3.4. ROESY Experiments

**Figure 10:**
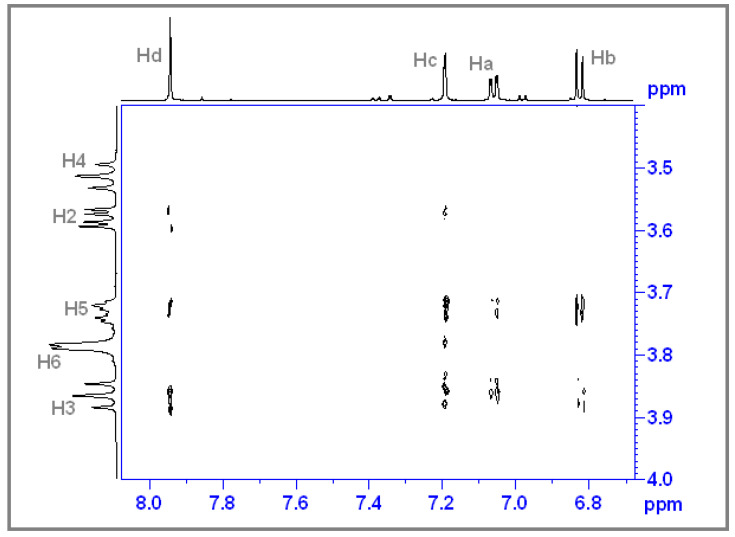
Expanded region of the ROESY spectrum of CHT:β-CD complex. [CHT] = 4 mM; [β-CD] = 6 mM.

A ROESY NMR spectrum being a two-dimensional (^1^H-^1^H), can prove the intermolecular interactions through space such as the non-covalent bonds between two different molecular species. Thus, being able to observe which specific protons from the two types of molecules present such interactions. As can be seen in Figure 10, the H3 and H5 protons inside the β-CD cavity interact with the Ha, Hb, Hc, and Hd protons belonging to the CHT molecule, which indicates that the CHT molecule is included inside the β-CD cavity with the aromatic ring, in agreement with our previous results on similar structures [8,31,43,44] and also in agreement with the theoretical results.

### 3.5. Evaluation of the Association Constant

The association constant was determined based on the same set of samples used to determine the stoichiometry of the CHT:β-CD complex which was found to be 1:1. With the help of the following equation, which is based on the variation of the chemical shift Δ*δ*^(i,j)^ depending on the concentration, the association constant can be determined [45]:

(2) Δδ(i,j)=Δδc(j)2[X]{[C]+1K−[([C]+1K)2−4[H](i)[G](i)]12}

with *i* being the sample number and *j* the investigated proton. The variation of chemical shifts:

$$\Delta \delta^{\left(i.j\right)}=\delta_{free}^{\left(i,j\right)}-\delta_{obs}^{\left(i,j\right)}$$

for a proton *j*, in a complexed _(*obs*)_ or free _(*free*)_ state.

(3) C=H+G

where *G* = guest and *H* = host

In order to calculate the association constant, a script developed by our group was used [46], based on an iteration procedure following specific algorithms, which takes into account the experimental values of the variations chemical shifts depending on the concentration, of the protons that show the most pronounced variations, both from the CHT molecule and H3 and H5 from β-CD.

Each iteration sets up a quadratic program which determines the direction of search and the loss function, until convergence.

(4) $$E=\sum_{i,j}(\Delta \delta^{\left(i,j\right)}-\Delta \delta_{calc}^{\left(i,j\right)}{)}^{2}$$

Using the procedure described above, an association constant was determined with the value K = 6.181 × 10^3^ M^−1^, with a correlation factor r = 0.991 and E = 3.656 × 10^−3^.

### 3.6. Molecular Docking

Cyclodextrins have found numerous applications in food, pharmaceutical, drug delivery, and chemical industries, as well as in agriculture and environmental engineering and are consequently produced in large quantities. Supramolecular inclusion compounds of cyclodextrins are consequently intensively studied and very diverse. Practically, cyclodextrins are already ingredients in 30 different approved medicines [47] and α-, β-, and γ-cyclodextrin are recognized to be generally safe by the U.S. FDA [48]. Having a hydrophobic interior and hydrophilic exterior, cyclodextrins form complexes with hydrophobic compounds, providing increased solubility and stability to the included drugs.

β-cyclodextrin, a cyclic heptasaccharide produced from starch by enzymatic conversion is the most used natural cyclodextrin. Because the internal cavity of β-CD has an appropriate dimension to include phenyl and/or thiazole moieties, in the actual study, we concentrated our attention on a close experimental and theoretical investigation of its complex with CHT which, as mentioned, we synthesized and previously studied in interaction with human serum albumin [22]. In this subchapter, we will present the theoretical molecular docking simulations performed with the goal of better understanding the interactions at a molecular level.

**Figure 11:**
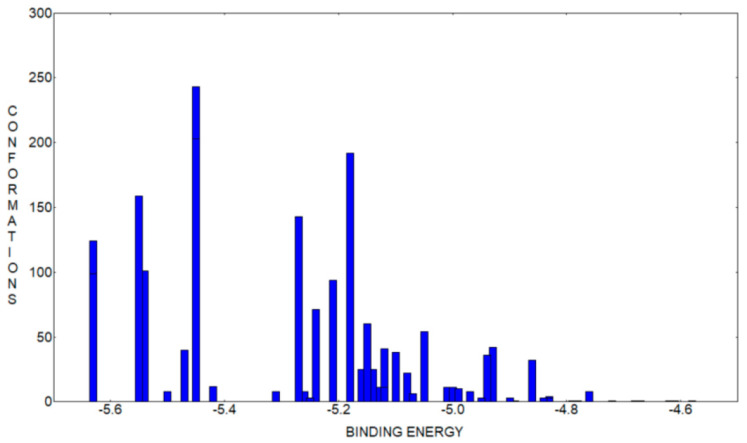
Histogram of the binding energy distribution to β-cyclodextrin of the cluster conformations of CHT.

Out of the mentioned 2000 docking runs, we obtained 51 distinct conformational clusters, using a root mean square deviation of 2.0 Å. In Figure 11 we have presented the histogram of the binding energy distribution of the identified clusters, the intensity representing the number of conformations belonging to each cluster. If we compare this relatively reduced number of clusters with more than 1000 obtained in our previous study, were we analyzed the inclusion compound of deferoxamine B chelator (DFOB) with β-CD [32], we can infer, from the beginning, that the conformational degrees of freedom of CHT vs. DFOB are drastically reduced. This fact can surely help and constrain our search to identify the most probable binding conformation but, as we will see in the following, even when we analyze small ligand molecules some strained conformations can be favored too, and this is most probably due to the approximations considered in our approach.

**Figure 12:**
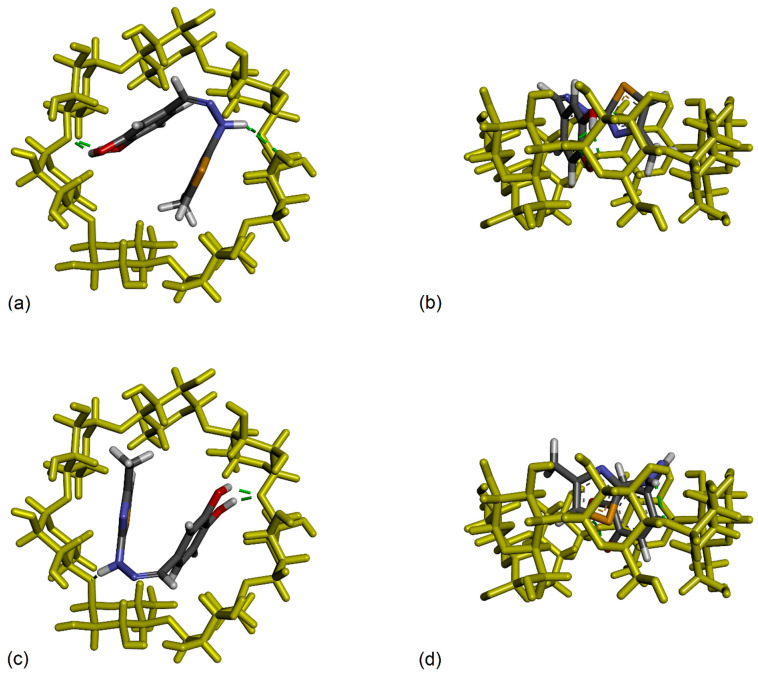
Inclusion compounds of CHT into the cavity of β-cyclodextrin as resulted from molecular docking calculations. (**a**,**c**) Secondary rim view and (**b**,**d**) side view of the leading clusters conformations having both an estimated binding energy of −5.63 kcal/mol. The cyclodextrin molecule was maintained fixed for a better comparison of these two similar conformations.

If we analyze the lowest binding energy conformations represented in Figure 11, appearing at −5.63 kcal/mol, which correspond to the strongest binding conformers, to our surprise we observed that we don’t have a unique most favorable conformation, but we have two clusters superposed, one with 99 members and the other with 124 members, both having the leading conformation with the same lowest binding energy. The mean binding energy value corresponding to these two clusters is also very similar, −5.10 kcal/mol vs. −5.21 kcal/mol, which means that, even if the conformations are practically different, they appear with a close probability. We will continue our analysis by taking a closer look at these two conformations. We represent them comparatively in Figure 12 presenting two views, a secondary rim view and a side view, for better visibility. Two important structural details can be remarked if we analyze these docking conformations. Firstly, we observe that the dihedral angle across the hydrazone group linking the two moieties of the CHT molecule was twisted in such a way that catechol and thiazole are nearly facing each other and, in these constrained conformations, the CHT molecule fits inside the β-CD cavity. Secondly, we observe that between the two conformations we don’t have a rotation symmetry only but mainly a reflection and rotation, because the sulfur atom of the thiazole group points outwards of the β-CD cavity, in one conformation, and in the other conformation, it is pointing inwards.

How can we rationalize these results? Firstly, we can understand that these packed conformations were favored by the docking algorithm because, in this way, not only the intermolecular interactions but also, the intramolecular π–π and π–sulfur CHT interactions can exist and contribute, consequently, to minimize the total energy of the complex. Here, we are aware that, in practice, such a constrained conformation probably has a short lifetime, mainly due to the dynamic interaction with the solvent molecules, which were not taken into account in our docking analysis. It is possible, therefore, as water molecules play a natural and definite role in the formation of the complex, other more relaxed conformations to be statistically favored. And, secondly, why not a simple rotation between these two conformations having practically the same binding energy, and we observe also the reflection? In our opinion this is due to the fact that the structure of β-CD used in our analysis and maintained rigid during our simulations, is not perfectly symmetric, being extracted, as mentioned, from an X-rays determined crystallographic inclusion compound with Mg^2+^ and Ca^2+^ salts of meclofenamic acid. It is known that atoms slightly move and adapt as a result of the interaction, and that the β-CD cavity has a small flexibility, which again was artificially ignored in our simulations. The crystallographic structure of the pure β-cyclodextrin cannot be perfectly symmetric too, because it depends on the strain in the macrocyclic ring of glucose subunits joined by α-1,4 glycosidic bonds, apart from the fact that, it presents hydrogen bonds with adjacent cyclodextrins and with some of the water molecules of crystallization. It is consequently surprising that, for our particular system, these two packed conformations, similar but different at the same time, turned out to have the same theoretical binding energy.

**Figure 13:**
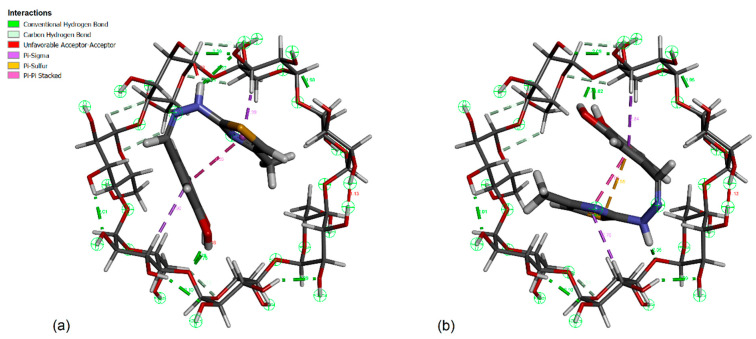
Close interactions of both conformations of CHT inside the β-cyclodextrin cavity. (**a**) 124 members cluster leading conformation, (**b**) 99 members cluster leading conformation.

To explore further and continue the analysis, in Figure 13, we present these two conformations where we additionally represented all types of close intra- and inter-molecular interactions. Here, we can observe, even visually, that the torsion angles across the hydrazone group slightly differ between the two conformations, practically with about 3.5°, and, also, that the catechol and thiazole rings are not perfectly parallel. If we were to attempt a comparison between the NMR experimental results and our actual theoretical calculations, we can observe that the most sensitive proton, presenting the largest chemical shift variation was the proton Hd belonging to hydrazone group and close to the catechol ring. Also, the next protons presenting large chemical shift variations were the proton Hc from catechol group and the proton He of the thiazole ring. All these experimental results converge to the conclusion that both rings of the CHT molecule are included in the β-CD cavity with the hydrazone proton, Hd, most involved in the interaction. Both of our theoretical structures, also considering the uncertainties due to the missing water, presented previously, can effectively confirm the experimental results.

## 4. Discussion

The scavenging of DPPH by the CHT-β-CD complex compared to free CHT is obviously delayed due to the complexation. It could be observed that there is a kinetics of release of CHT from the complex over time, compared to its free form. At the lowest concentration in the assay, the effect is almost linear in time, but at the average concentration and the highest concentration, it can be observed that the antiradical effect is more intense in the first part of the experiment (higher slope), after which the effect has a greater increase slow (smaller slope). It could be said that the graph would have an asymptotic trend.

The value of the CHT-β-CD association constant determined by both methods (ITC and NMR) is high if we compare it with the values obtained for other β-CD inclusion complexes investigated by us (procaine, benzocaine, tolmetin, flurbiprofen, 3-carboxy-1-[(2-phenyl-1,3-thiazol-4-yl) methyl]pyridin-1-ium iodide), having values between 10^2^ and 10^3^ M^−1^.

From a theoretical point of view, surely, a molecular dynamics investigation where the solvent molecules are explicitly considered and where all atoms can move and relax as a result of the interactions, can refine and improve these minimal conformations but this analysis exceeds the purpose of this study being too computationally intensive.

## 5. Conclusions

The encapsulation of CHT into β-CD was studied by ITC in combination with the spectroscopic method NMR and molecular docking simulation. Based on the presented studies, it can be concluded that the CHT inclusion complex with β-CD was formed in a molar ratio of 1:1. ITC measurements of CHT–β-CD complex indicate spontaneity (ΔG < 0) of the ligand–receptor process. The formation of the complex is controlled by enthalpy (ΔH < 0) and entropic (ΔS > 0) effects. Thus, the inclusion complexation of CHT: β-CD is predominantly governed by hydrophobic interactions driven by entropy energy. The association constants (K) obtained from both the ^1^H NMR and ITC experiments for CHT complexation with β-CD are in the same order of magnitude.

The CHT: β-CD complex in aqueous solution has been studied by ^1^H NMR spectroscopy. An analysis of our data by the continuous variation method indicates that the inclusion occurs and the complex has 1:1 stoichiometry. The ROESY NMR experiment highlights intermolecular interactions through space between the H3 and H5 protons inside the β-CD and the Ha, Hb, Hc, and Hd protons belonging to the CHT molecule, which indicate that the CHT molecule is included inside the β-CD cavity with the aromatic ring. The association constant K obtained by ^1^H NMR and ITC are in agreement and both methods sustain a 1:1 stoichiometry.

The antiradical activity of CHT when included in a complex with β-CD is extended in time, compared to free CHT.

As a result of the docking analysis, we identified two possible conformations of the inclusion compound which agree with the largest chemical shift variations of the protons, corresponding to the CHT molecule, obtained from NMR experiments. We observed that these rough but fast simulations are full of insights and contain rather precise details. Molecular dynamics simulations have the ability to improve these estimations because, in this case, all atoms of the host and guest molecules can freely move and the solvent molecules are considered explicitly.

## Figures and Tables

**Table 1:** The thermodynamic parameters of CHT/β-CD.

N	K_a_ (M^−1^)	ΔH (kJ mol^−1^)	TΔS (kJ mol^−1^)	ΔG (kJ mol^−1^)
1.098	3.92 × 10^3^	−2.36	18.13	−20.5

**Table 2:** Chemical shifts of CHT protons in the free state (ppm).

Proton	Ha	Hb	Hc	Hd	He	Hf
δ	7.029	6.801	7.137	7.843	6.387	2.145

## Data Availability

The data presented in this study is available in the article.
